# Comparative Study on Recognition Models of Black-Odorous Water in Hangzhou Based on GF-2 Satellite Data

**DOI:** 10.3390/s22124593

**Published:** 2022-06-17

**Authors:** Zhifeng Yu, Qiyu Huang, Xiaoxue Peng, Haijian Liu, Qin Ai, Bin Zhou, Xiaohong Yuan, Meihong Fang, Ben Wang

**Affiliations:** 1 Institute of Remote Sensing and Earth Sciences, School of Information Science and Technology, Hangzhou Normal University, Hangzhou 311121, China; yu@hznu.edu.cn (Z.Y.); 2019210214026@stu.hznu.edu.cn (Q.H.); 2020111008047@stu.hznu.edu.cn (X.P.); haijian@hznu.edu.cn (H.L.); 2017210214002@stu.hznu.edu.cn (Q.A.); zhoubin@hznu.edu.cn (B.Z.); yuanxh@hznu.edu.cn (X.Y.); melodymhfang@hznu.edu.cn (M.F.); 2 Zhejiang Provincial Key Laboratory of Urban Wetlands and Regional Change, Hangzhou 311121, China

**Keywords:** black-odorous water, GF-2, remote sensing

## Abstract

To improve the ability of remote sensing technology in recognizing black-odorous water bodies in Hangzhou, this study analyzed the typical spectral characteristics of black-odorous water in Hangzhou based on measured spectral data and water quality parameters, including the transparency, dissolved oxygen, oxidation reduction potential, and ammonia nitrogen. The single-band threshold method, the normalized difference black-odorous water index (NDBWI) model, the black-odorous water index (BOI) model, and the color purity on a Commission Internationale de L’Eclairage (CIE) model were compared to analyze the spatial and temporal distribution characteristics of the black-odorous water in Hangzhou. The results showed that: (1) The remote sensing reflectance of black-odorous water was lower than that of ordinary water, the spectral curve was gentle, and the wave peak shifted toward the near-infrared direction in the wavelength range of 650–850 nm; (2) Among the aforementioned models, the normalized and improved normalized black-odorous water index methods had a higher accuracy, reaching 87.5%, and the threshold values for black-odorous water identification were 0.14 and 0.1, respectively; (3) From 2015 to 2018, the quantity of black-odorous water in the main urban area of Hangzhou showed a decreasing trend, and black-odorous water was mainly distributed in the Gongshu District and tended to appear in narrow rivers, densely populated areas, and factory construction sites. This study is expected to be of great practical value for the rapid tracking and monitoring of urban black-odorous water by using remote sensing technology for future work.

## 1. Introduction

Black-odorous water not only destroys urban water ecosystems, affects the image of a city, and brings poor visual experience to the masses, but also hinders urbanization and economic development. According to the Action Plan for Prevention and Control of Water Pollution by The State Council in 2015, the quantity of black-odorous water bodies in built areas of cities at the prefecture level and above should be controlled within 10% by 2020, and ultimately eliminated by 2030 [1]. In a press conference held by the Ministry of Ecology and Environment in 2021, this work was said to have achieved phased results. By the end of 2020, the proportion of black-odorous water in built areas of prefecture-level and above cities in China reached 98.2% [2]. To realize a smooth execution of the 2030 plan, the treatment of black-odorous water bodies should be fast tracked.

Hangzhou is a typical water village in the south of the Yangtze River: the water environment of urban rivers is seriously polluted. Faced with the problem of black-odorous water in urban areas, Zhejiang Province, as a pilot of national ecological civilization construction, put forward a positive response to “five water work” and “long river cruise river system” policy, and formulated the Action Plan for Prevention and Control of Water Pollution in Hangzhou [3]. It provides concrete measures for the treatment of black-odorous water bodies. Under the combined efforts of various parties, Hangzhou has achieved remarkable results in the treatment of black-odorous water. To consolidate the treatment achievements and comprehensively eliminate such water bodies, it is necessary to strengthen the treatment procedure. Water sampling and chemical analysis are typically used to monitor black-odorous water bodies [4]. The Guideline for Urban Black and Odorous Water Treatment [5], issued by the Ministry of Housing and Urban-Rural Development of the People’s Republic of China, clearly indicates that the evaluation indexes of urban black-odorous water bodies include transparency, dissolved oxygen, potential, and ammonia nitrogen, and can be used to determine whether the water is black and odorous [6]. With the development of remote sensing technology, satellite remote sensing images are being used to monitor black-odorous water bodies, providing a technical means of monitoring.

Domestic researchers have used remote sensing to study the spectral curve of black-odorous water. With the increase in high-resolution satellite data in China, researchers have used domestic high-resolution image data, water quality parameters, and spectral characteristic recognition models to construct algorithms for black-odorous water; moreover, satellite sensors have been tested for remote sensing monitoring, classification, and recognition of black-odorous water bodies. Li et al. [7] extracted and analyzed lake flooding along the western coast of Taihu Lake through Landsat-7 ETM+ images, and summarized the remote sensing spectral signal characteristics of lake flooding phenomena caused by blue algae, which often leads to black-odorous water in cities, to identify the lake flooding. Zhu et al. [8] used the GF-1 WFV sensor to invert the water quality parameters and found that this sensor has a high application potential in water quality detection and the inversion of water quality parameters. Wang et al. [9] monitored the water quality in Chao Lake through the HJ-1A satellite and confirmed the suitability of the satellite images for water quality monitoring. Similar studies have confirmed the potential of remote sensing satellite images for water quality monitoring. Jin et al. [4] used the GF-2 satellite for the water quality parameter inversion for black-odorous water bodies in Beijing, and found that this satellite has good application potential in identifying black-odorous water bodies in narrow urban river channels. Further, in the application of remote sensing recognition of urban black-odorous water bodies in China, according to the characteristics of different sensors and differences in remote sensing images, many experts and scholars have proposed various remote sensing recognition algorithms for urban water quality. For example, Yao et al. [10] proposed a BOI model to identify the black-odorous water in Shenyang by sampling and analyzing the spectrum and water quality parameters of the water body. Wen et al. [11,12] used a Gaofen-2 satellite (hereinafter referred to as GF-2) image to study the black-odorous water body in Nanjing and constructed a recognition algorithm for urban black-odorous water bodies based on chroma and other multiple indicators, and one based on the feature band. Gi et al. [13] constructed the HCI black-odorous water model discriminant index based on GF-1 and GF-2 images and the reflectance spectral characteristics of black-odorous water and general water. Qian et al. [14] used a GF-2 image to analyze two different types of black-odorous water bodies and general water bodies; they proposed the CIE color purity algorithm, which was found to be superior to the ratio algorithm. Qi et al. [15,16] constructed a remote sensing classification index, called the BOCI model, for urban black-odorous water bodies based on the difference in the reflectance spectra between ordinary water body, mild black-odorous water body, and severe black-odorous water body. Lu [17] modified the Nemerow index method using the classification of the black-odor grade of water in the Guide, so as to achieve a continuous distribution of the black-odor grade evaluation results. They verified the accuracy of their respective algorithms, concluded that they met the actual requirements, and evaluated their applicability. In relative terms, the urban water quality monitoring system employed abroad is relatively mature and complete. Foreign researchers have used empirical models, bio-optical models, neural network models, and other methods to remotely monitor the water quality [18]. Olmanson et al. [19] established a transparency database for Minnesota lakes through Landsat images, and, through evaluations, proved that remote sensing images can accurately provide comprehensive water quality parameter information with time and space coverage. Previous researchers have built algorithmic models to sort black water bodies by processing remote sensing images; however, black-odorous water contains not only black water bodies, but also odorous water bodies, which cannot be distinguished by remote sensing image processing. Hence, by analyzing the spectrum of common odorous water bodies in cities, such as odorous water bodies due to excessive algae, represented by excessive chlorophyll concentration, or the odor due to a certain amount of colored dissolved organic matter, the spectral characteristics can be summarized and finally used to identify odorous water by remote sensing. Thus, the following results also have a certain reference significance for the discrimination of smelly water bodies. Dekker and Peters [20] monitored the water quality of the eutrophic lake Loosdrecht in the Netherlands through Landsat TM image data in different periods; they concluded that the suspended matter concentration and chlorophyll concentration in the water body were strongly correlated with TM2. By using Landsat-8 and Sentinel-2 images, Kutser et al. [21] found that compared with Sentinel-2, Landsat-8 band was unsuitable for detecting the two peaks in the reflection spectra of many lakes. Tassan [22] improved the correlation between the water quality parameters and remote sensing data by taking logarithms of the band combinations or water quality parameters. Malthus et al. [23] used hyperspectral remote sensing images to analyze lakes with different nutrient conditions and determine bands with good correlation with the sediment concentration. Roeck et al. [24] used IKONOS images to compare and analyze the accuracy of multilevel classification and nonhierarchical classification methods in estimating the parameters of black-odorous water bodies. Xian et al. [25] effectively extracted black-odorous water bodies in Seattle, Las Vegas, and Tampa Bay using high-resolution satellite images and UAV images. Tassan et al. [26] used SeaWiFS to find the optimal band for estimating the total suspended matter concentration and build an algorithm model.

Currently, the remote sensing water-color recognition method and the urban black-odorous water body recognition model have high accuracy in practical applications; however, some drawbacks remain. For example, the threshold selection of each algorithm model is different, the application is unstable in areas outside the study area, the effect of water-body extraction becomes worse, and there is sometimes a significant error when the algorithm is applied to different satellites. Therefore, how to select the optimal threshold or automatically extract the water bodies is a major problem of each algorithm model [27].

In this study, based on the Gaofen-2 satellite image and the measured water spectrum of the main river system in Hangzhou, four kinds of black-odorous water body recognition models are used to identify the water bodies in Hangzhou city. The accuracy of the inversion results is tested one by one using the measured water body data, and the applicability of various water body recognition algorithms is compared. Finally, the black-odorous water body recognition model with the highest applicability is obtained, and the inversion results are briefly analyzed. At the same time, reflecting on the possible errors of the research and the explorable direction of the future research.

## 2. Study Area and Data

### 2.1. Study Area

**Figure 1:**
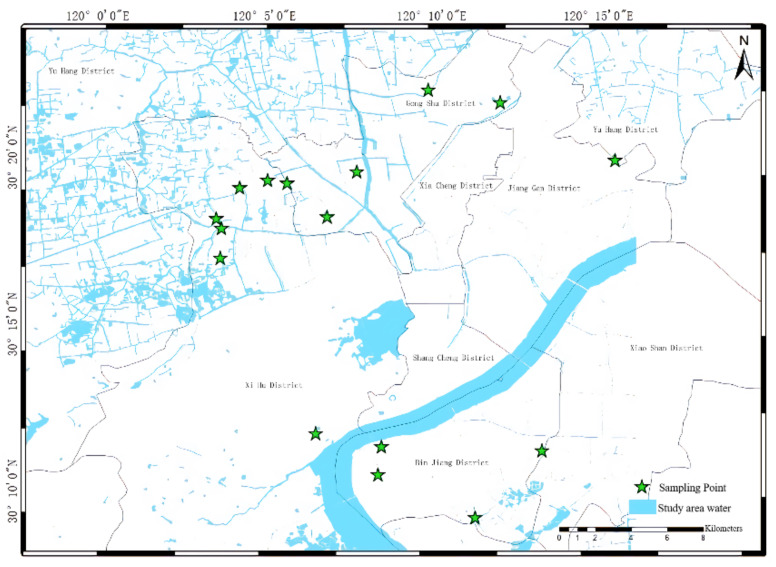
Measured water points distribution.

Hangzhou is located in the southeast coast of the northern part of Zhejiang Province (Figure 1). It has a subtropical monsoon climate with four distinct seasons and abundant rainfall. The city is at the lower reaches of the Qiantang River and the southern end of the Beijing-Hangzhou Grand Canal. The city has a dense river network, with a total of 3223 river channels, including 470 river channels around the city and 2753 river channels outside the city [28]. Although there are many rivers, the water environmental capacity is low, and the self-purification capacity of the water bodies is poor. According to the annual Hangzhou environmental bulletin, published by Hangzhou Environmental Protection Bureau, the proportion of 52 city-controlled sections in Hangzhou reaching or better than Class III standards from 2015 to 2018 were 85.1%, 85.1%, 88.5%, and 92.3%, respectively (http://www.hangzhou.gov.cn, accessed on 5 July 2016, 5 June 2017, 11 June 2018, 5 June 2019, respectively). It can be seen that the water quality of the whole city is improving steadily, but in view of the frequent occurrence or recurrence of urban black-odorous water, the rivers may turn black and odorous if prevention, discharge, and treatment measures are inadequate.

### 2.2. Field Data

In order to obtain the spectral data of the water body, this study refers to Tang Junwu’s measurement method (Above-Water Method) [29], using the American HH2 (HandHeld 2) hand-held ASD spectrometer and 30% reflectivity standard board. We obtains the water body spectral data of sampling points, removes the abnormal spectra with large deviation in each observation point, and calculates the remote sensing reflectance of the remaining spectral data (the formula is as follows). The water quality parameter data, including dissolved oxygen, ammonia nitrogen, and oxidation potential, are obtained by multi-parameter water quality meter. After setting the water quality parameters to be tested, the corresponding water quality parameter information can be obtained by turning on the automatic measurement mode. The transparency information of the water body is measured by transparency meter, and the average value of twice measurement is taken as the transparency value of water body.

(1) $$R_{rs}=\frac{\left(L_{w}-\rho L_{sky}\right)\rho_{p}}{\pi E_{s}}$$

here, $R_{rs}$ is the remote sensing reflectance, $L_{w}$ is the radiance of the water body measured by a spectrometer, $L_{sky}$ is the radiance of sky diffuse scattering measured by a spectrometer, $E_{s}$ is the radiance of the standard plate measured by a spectrometer, and $\rho_{p}$ is the reflectance of the standard plate that has been strictly calibrated. ρ is the reflectance of the air–water boundary facing sky light; in this study, ρ was taken as 0.022.

To analyze the remote sensing reflectance of the treated water body, the spectral differentiation method was adopted in this study. The spectral differentiation technology can eliminate partial atmospheric effects and environmental background influences and enhance subtle changes in the spectral curves on the slope [30]. The first-order spectral differentiation formula is as follows:

(2) $$\rho^{\prime }\left(\lambda_{i}\right)=\left[\rho \left(\lambda_{i+1}\right)-\rho \left(\lambda_{i-1}\right)\right]/2\Delta \lambda$$

In the formula, $\rho^{\prime }\left(\lambda_{i}\right)$ is the first-order differential value at wavelength $\lambda_{i}$, which is the wavelength of each band, and Δλ is the interval between adjacent bands.

Based on the remote sensing reflectance of the water body and spectral response function of the GF-2 remote sensing image, the equivalent reflectance of the satellite band is calculated using the integral formula. Since the general spectral response function is discrete, the band equivalent is the weighted average of the incident spectral data according to the wavelength. The calculation formula is as follows:

(3) $$L_{Normalization}=\frac{\int_{\lambda_{min}}^{\lambda_{max}}L\left(\lambda \right)f\left(\lambda \right)d\lambda }{\int_{\lambda_{min}}^{\lambda_{max}}f\left(\lambda \right)d\lambda }$$

In the formula, $L_{Normalization}$ is normalized to the equivalent reflectance value of the band, *L*(*λ*) is the remote sensing reflectance of the water at each sampling point, and *f*(*λ*) is the spectral response function of the GF-2 remote sensing image. $\lambda_{min}$ and $\lambda_{max}$ are the wavelength range of the spectral response function

In this study, the parameters of some urban river channels in the main urban area of Hangzhou were measured in the field on 27–28 July 2018 and on 23–24 March 2019. Among them, the data from eight sampling points were used to verify the accuracy of the recognition model for black-odorous water bodies, and data from the other sampling points were used to establish a black-odorous water body recognition model.

### 2.3. High-Resolution Image Data

GF-2 remote sensing image data for the years 2015, 2016, and 2018 were obtained from China Resources Satellite Application Center (http://www.cresda.com/CN/, accessed on 2 August 2015, 16 March 2016 and 16 March 2016, respectively). The GF-2 satellite is equipped with a 1 m panchromatic and 4 m multispectral high-resolution camera, with a sub-satellite point spatial resolution of 0.8 m, a ground width of 45 km, a real-time GPS positioning accuracy of 10 m, and a post-processing accuracy of 50 cm. It has a submeter spatial resolution and a high positioning accuracy. Data support will be provided for monitoring the water conservancy, agriculture, forestry, disaster reduction and relief, land and resources, and other sectors. The GF-2 remote sensing image adopted in this study covers the research area with less cloud cover, which can be used for comparing and verifying the identification models of the black-odorous water in Hangzhou and for studying its spatial and temporal distribution. Table 1 and Table 2 present the imaging parameters.

The pretreatment of the GF-2 image mainly includes six steps: radiometric calibration, atmospheric correction, orthography correction, image fusion, image clipping, and image mosaic. Radiometric calibration is to eliminate the error of the sensor itself and convert the DN value into radiance value. The specific formula is shown in Equation (4). Atmospheric correction is to eliminate the influence of atmosphere and convert the radiation brightness value into the actual surface reflectance. The FLAASH atmospheric correction method in ENVI was adopted in this study. Orthophoto correction is to eliminate the influence of terrain and generate a plane orthophoto image. Image fusion refers to the fusion of the GF-2 panchromatic and multispectral images; the Gram-Schmidt Pan Sharpening fusion method in ENVI was adopted in this study.

(4) L=gain×DN+offset

here, *L* is the brightness value, *DN* is the pixel gray value, and *offset* is the offset quantity.

To effectively extract black-odorous water bodies, the water in the study area was masked. The water in the GF-2 remote sensing image after pretreatment was extracted using the NDWI. The calculation formula is as follows:

(5) $$NDWI=\frac{R_{rs}\left(Green\right)-R_{rs}\left(NIR\right)}{R_{rs}\left(Green\right)+R_{rs}\left(NIR\right)}$$

$R_{rs}\left(Green\right)$ and $R_{rs}\left(NIR\right)$ represent the green and near-infrared band remote sensing reflectance of the water body measured at the sampling point, respectively.

When using the above formula to extract the water boundary in the image, the threshold is 0. The accuracy of the confusion matrix and Kappa coefficient of the final water-body extraction results all meet the expected requirements.

### 2.4. Model Introduction

#### 2.4.1. Single-Band Threshold Method

The remote sensing reflectance of the black-odorous water body is lower than that of the ordinary water body, and the difference between the second band of the GF-2 remote sensing image and ordinary water body is the highest. Therefore, the remote sensing reflectance value of the second band is used to identify the black-odorous water body.

(6) $$0\leq R_{rs}\left(Green\right)\leq T$$

$R_{rs}\left(Green\right)$ is the remote sensing reflectance after atmospheric correction in the second band of the GF-2 remote sensing image, and T is a constant.

#### 2.4.2. NDBWI

The spectral curve of the black-odorous water is different from than that of the average water flat curve. For the GF-2 remote sensing images from the second wave to the third band, the black-odorous water remote sensing reflectance relative to the general water drop is small, but generally has a high water remote sensing reflectance; therefore, the second and the third band remote sensing reflectance difference and the ratio of the identification of the black water can be expressed as follows:

(7) $$\begin{array}{l} NDBWI=\frac{R_{rs}\left(G\right)-R_{rs}\left(R\right)}{R_{rs}\left(G\right)+R_{rs}\left(R\right)}\\ T_{1}\leq NDBWI\leq T_{2} \end{array}$$

$R_{rs}\left(R\right)$ and $R_{rs}\left(G\right)$ respectively represent the remote sensing reflectance values of the third and second bands of the GF-2 image after atmospheric correction, respectively. $T_{1}$ and $T_{2}$ are constants.

#### 2.4.3. BOI

From the second band to the third band of the GF-2 image, the remote sensing reflectance of the black-odorous water slightly decreased compared with that of the general water body. Therefore, the difference between the green and red band remote sensing reflectance values was taken as the numerator, and the sum of the remote sensing reflectance values of the three bands was taken as the denominator.

(8) $$\begin{array}{l} BOI=\frac{R_{rs}\left(G\right)-R_{rs}\left(R\right)}{R_{rs}\left(G\right)+R_{rs}\left(R\right)+R_{rs}\left(B\right)}\\ T_{1}\leq BOI\leq T_{2} \end{array}$$

$R_{rs}\left(R\right)$, $R_{rs}\left(G\right)$, and $R_{rs}\left(B\right)$ represent the remote sensing reflectance values of the third, second, and first bands of the GF-2 remote sensing image after atmospheric correction, respectively. *T*_1_ and *T*_2_ are constants.

#### 2.4.4. CIE

The distribution and characteristics of different ground objects in remote sensing images can be expressed by different colors, that is, each ground object has its specific color, so as to facilitate differentiation in remote sensing images. Black-odorous water has a lower reflectivity and darker color than ordinary water, which can be used to identify such bodies.

(9) $$\begin{array}{l} X=2.7689R+1.7517G+1.1302B\\ Y=1.0000R+4.5907G+0.0601B\\ Z=0.0000R+0.0565G+5.5934B \end{array}$$

(10) $$\begin{array}{l} x=\frac{X}{X+Y+Z}\\ y=\frac{Y}{X+Y+Z}\\ T_{1}\leq \lambda \leq T_{2} \end{array}$$

R, G, and B are the third, second, and first bands corresponding to the equivalent reflectance of the GF-2 remote sensing image; X, Y, and Z are the three stimulus values of the ground object; *x* and *y* are the chromaticity coordinates of the ground object; λ is the main wavelength corresponding to the ground chromaticity coordinates; $T_{1}$ and $T_{2}$ are constants.

## 3. Results and Discussion

### 3.1. Analysis of the Typical Spectral Characteristics of Black-Odorous Water Body

#### 3.1.1. Analysis of Reflectance Spectral Characteristics

**Figure 2:**
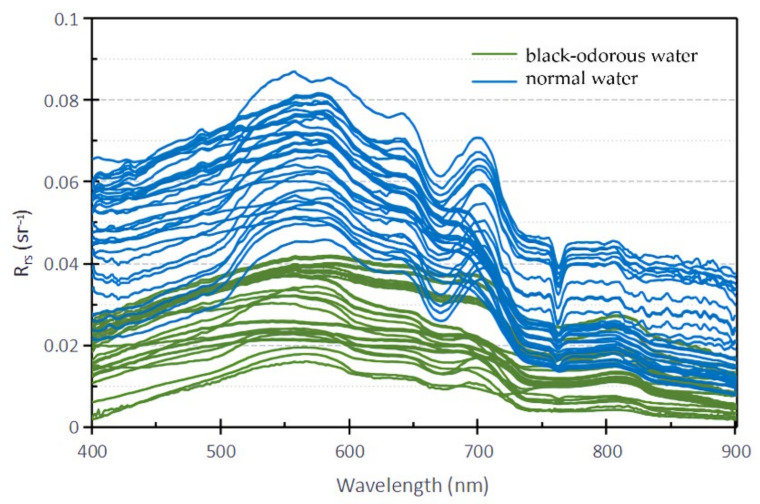
Reflectance spectra of black-odorous water and normal water.

To obtain the reflectance spectral difference between the black-odorous water body and ordinary water body in Hangzhou, the studied rivers were divided into black-odorous water body and ordinary water body in accordance with the guidelines for the treatment of black-odorous water bodies [5], and their remote sensing reflectance values were calculated. Mapping was made based on the classification results, and the remote sensing reflectance spectra of the black-odorous water bodies and general water bodies were compared (Figure 2).

Overall, the spectral curves of the reflectance of the black-odorous water bodies and ordinary water bodies have two evident peaks. However, the reflectance value of the black-odorous water body is lower than that of the general water body. The reflectance value range of the black-odorous water body is 0.00–0.04 sr^−^^1^, while that of the general water body is 0.00–0.10 sr^−1^. In addition, the rising speed of the two peaks in the reflectance curve of the black-odorous water is different from that of the two peaks in the reflectance curve of the ordinary water. The rising speed of the first and second wave peaks in the reflectance curve of the black-odorous water is lower than that of the first and second wave peaks in the reflectance curve of the general water.

Locally, the positions of the first wave peak in the remote sensing reflectance curves of the calculated black-odorous water body and general water body are similar; however, the positions of the second wave peak and the first wave trough are different. The wavelength of the second peak and the first trough in the remote sensing reflectance curve of the black-odorous water is higher than that of the second peak and the first trough in the remote sensing reflectance curve of the general water. From the specific data shown in Figure 2, the first wave peaks in the reflectance curves of the black-odorous water and general water are distributed in the wavelength ranges of 550–580 nm and 550–570 nm, respectively; the second wave peaks are located near 800 and 700 nm, respectively. The first trough of the reflectance curve of the black-odorous water is located near 740 nm, while the first trough of the general water is near 670 nm.

Therefore, apart from the remote sensing reflectance value, the wavelength range of the second wave peak in the reflectance curve and the rising speed of the first wave trough and wave peak can be used to distinguish the black-odorous water body from the general water body. In a remote sensing image, the difference in the rising speeds of the wave peaks can be reflected by blue and green wavelengths. Therefore, when using remote sensing technology to identify and monitor black-odorous water bodies, algorithms related to the blue, green, and red bands can be constructed for the analysis.

#### 3.1.2. Spectral Differential Analysis

**Figure 3:**
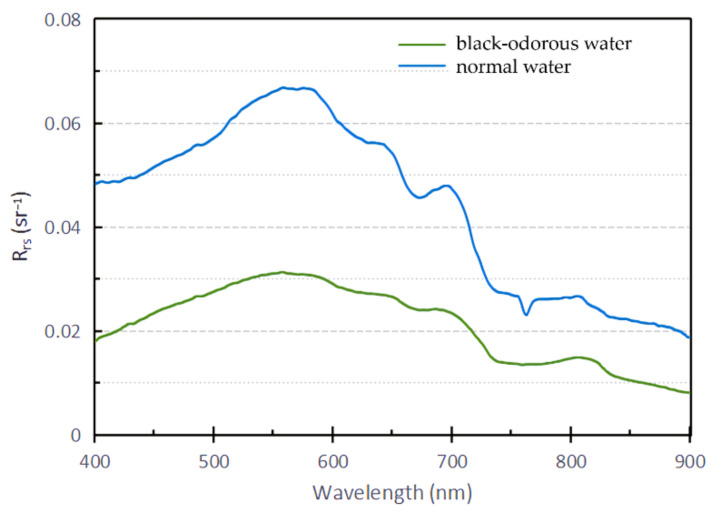
Comparison of black-odorous water with normal water.

The spectral differential technique is a technique that uses a mathematical method to deal with the spectral reflectance curve. To partially eliminate the influence of atmospheric effect and environmental background, this study conducted first-order differential treatment on the reflectivity curves of the black-odorous water and general water, and further analyzed the differences in their spectral characteristics. First, the average reflectance of all the collected black-odorous water bodies and general water bodies was taken, and the first-order differential formula was used to process the average value, as shown in Figure 3.

As shown in Figure 3, the remote sensing reflectance of the general water body decreases significantly at 590 nm and presents a shoulder at 642 nm, with a trough at 680 nm and a peak at 700 nm. The remote sensing reflectance of the black-odorous water decreases gradually in the wavelength range of 550–700 nm.

**Figure 4:**
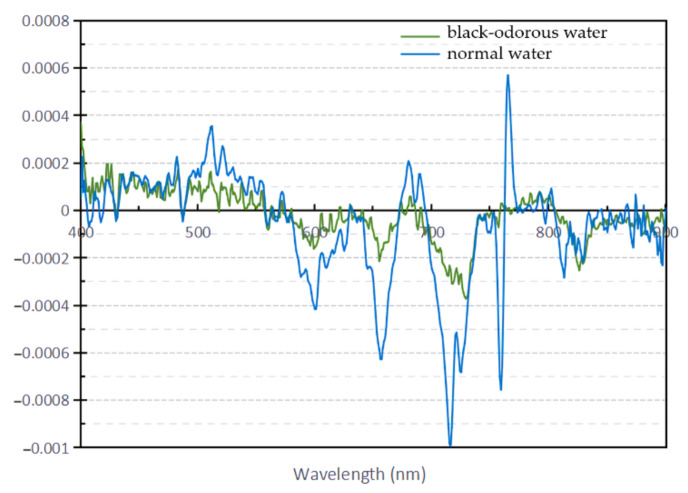
Comparison of the first derivative between the average spectrum of black-odorous water and the average spectrum of normal water.

The first derivative of the reflectance spectrum is positive, indicating that the reflectance spectrum shows an upward trend. A negative value indicates that the reflectance spectrum has a downward trend. A value of 0 indicates a reflection peak or valley in the spectral reflectivity curve. Figure 4 shows that the reflectance spectrum of the general water body varies significantly. The first derivative of the reflectance spectrum of the black-odorous water is higher than that of the reflectance spectrum of the ordinary water. The wavelength range of the first derivative of the reflectance spectrum of the black-odorous water at 0 is greater than that of the first derivative of the reflectance spectrum of the ordinary water, indicating that the range of peaks and troughs in the former is greater than that of the peaks and troughs in the latter. Combining the spectral reflectivity curves of the two water bodies to analyze the first derivative, it can be seen that the wavelengths of the first peak in the two cases are the same; however, the reflectivity curve of the black-odorous water has a wider first peak than that of the general water. After the first wave peak, the reflectance curve of the black-odorous water shifted to the long-wave direction compared with that of the ordinary water.

#### 3.1.3. Cause Analysis

Water quality parameters and algae are factors causing the spectral characteristics of black-odorous water to be different from those of ordinary water. Most algae contain chlorophyll. Due to the absorption of chlorophyll, the remote sensing reflectance of general water drops sharply at 650 nm and has a trough at 680 nm. Because of chlorophyll reflection, the remote sensing reflectance of the general water body has a peak at 700 nm. However, the black-odorous water has a low dissolved oxygen content due to water pollution (Table 3) and less algae, and its remote sensing reflectance has no characteristics similar to the chlorophyll spectral curve.

According to Gitelson [31], suspended substances not only affect the form of the reflection peak of the water body, but also shift the reflection peak wavelength to the long-wave direction. Therefore, the spectral curve of the black-odorous water body shows a trend in which the wave peak moves to the near-infrared direction in the wavelength range of 650–850 nm.

### 3.2. Black-Odorous Water Identification Model

**Figure 5:**
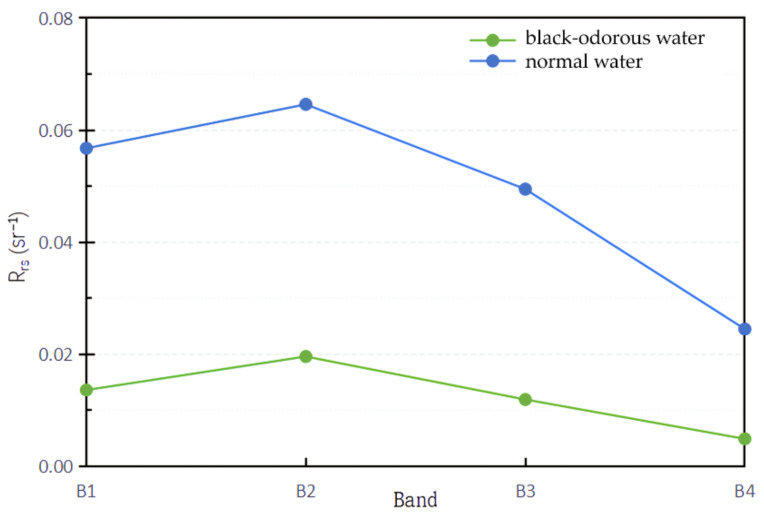
GF-2 simulation comparison of the mean reflectance of black-odorous water and general water.

Based on the analysis of the typical spectral characteristics of the black-odorous water body (Figure 5), the single-band threshold method, NDBWI, BOI, and CIE were used to identify the black-odorous water body.

#### 3.2.1. Recognition Model

(1) Single-band threshold method

**Figure 6:**
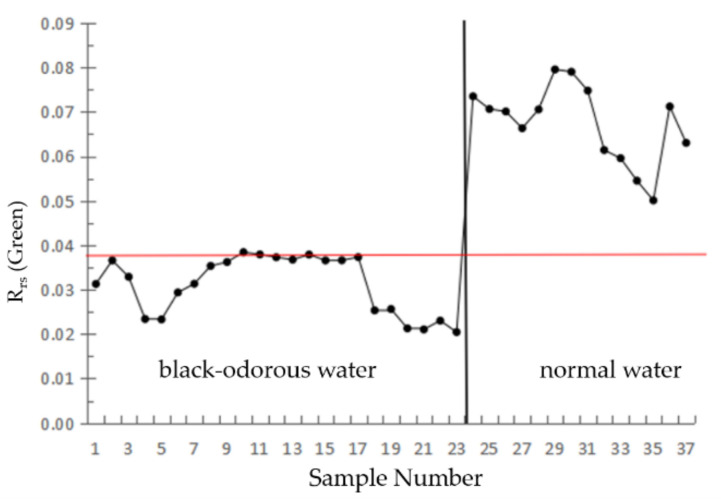
Single-band threshold method.

Figure 6 shows the remote sensing reflectance values of the modeling data of the black-odorous water body and general water body in the second band, and *T* = 0.038 sr^−1^ is selected by analysis.

(2) NDBWI

**Figure 7:**
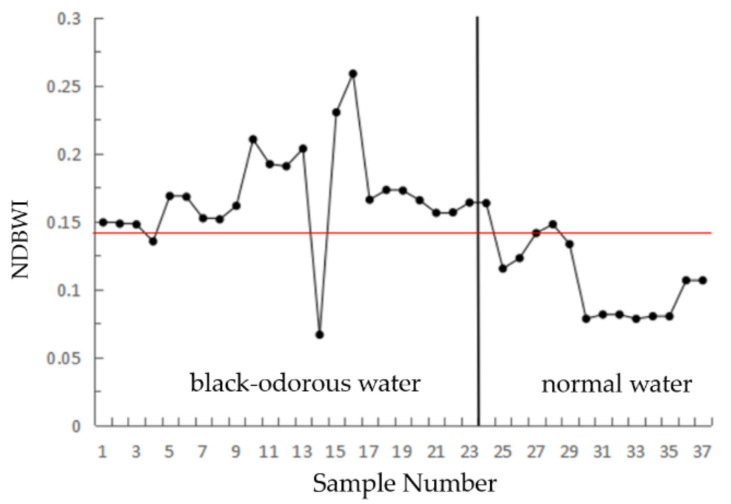
Normalized black-odorous water index.

Figure 7 shows the ratio of the remote sensing reflectance difference and the sum of the modeling data of the black-odorous water body and general water body in the second and third bands, where *T*_1_ = 0.140 and *T*_2_ = 0.250 after the analysis.

(3) BOI

**Figure 8:**
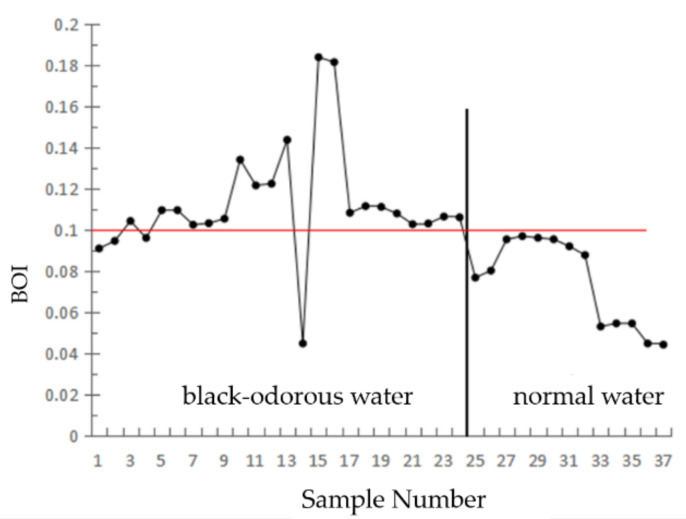
Normalized black-odorous water index after correction.

Figure 8 shows the ratio of the difference in the remote sensing reflectance of the modeling data of the black-odorous water body and general water body in the second and third bands to the sum of the remote sensing reflectance values of the first, second, and third bands, where *T*_1_ = 0.100 and *T*_2_ = 0.185 after the analysis.

(4) CIE

**Figure 9:**
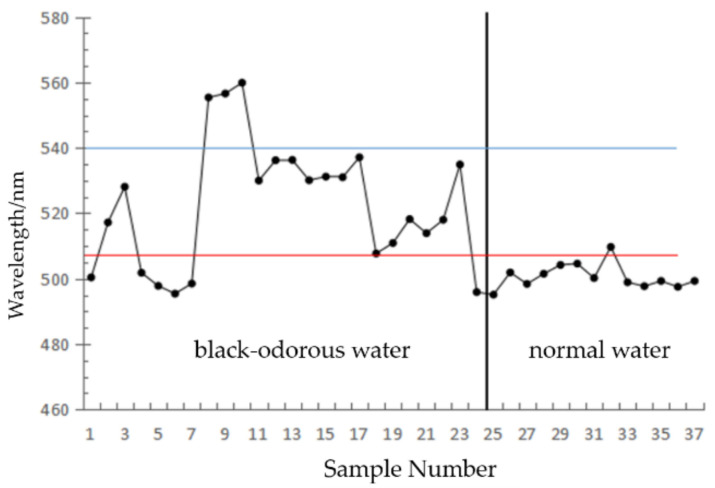
CIE colorimetry.

Figure 9 shows the modeling data of the black-odorous water body and general water body. The main wavelength was obtained on the basis of the remote sensing reflectance values of the first, second, and third bands. After the analysis, *T*_1_ = 507 nm and *T*_2_ = 540 nm were selected.

#### 3.2.2. Precision Analysis

The actual water quality of the verification samples was compared with the result of the identification algorithm, and the number of correctly identified verification samples was obtained. The accuracy of the four identification models of the black-odorous water was compared and analyzed (Table 4).

(11) $$\mathrm{Recognition}\;\mathrm{accuracy}=\frac{N_{\mathrm{Correct}\;\mathrm{identification}}}{N_{\mathrm{Total}}}\times 100\%$$

$N_{\mathrm{Correct}\;\mathrm{identification}}$ is the number of validation samples that can correctly identify black-odorous water after the identification model is adopted, and $N_{\mathrm{Total}}$ is the total number of validation samples that can correctly identify black-odorous water after the identification model is adopted.

P_1_ is located at Xitang River, P_2_ at Dianfang River, P_3_ at Fengjia River, P_4_ at Houhenggang River, P_5_ at Dongchen River, P_6_ at Fujiachi River, P_7_ at Yuhangtang River, and P_8_ at Nanhuanggang.

**Figure 10:**
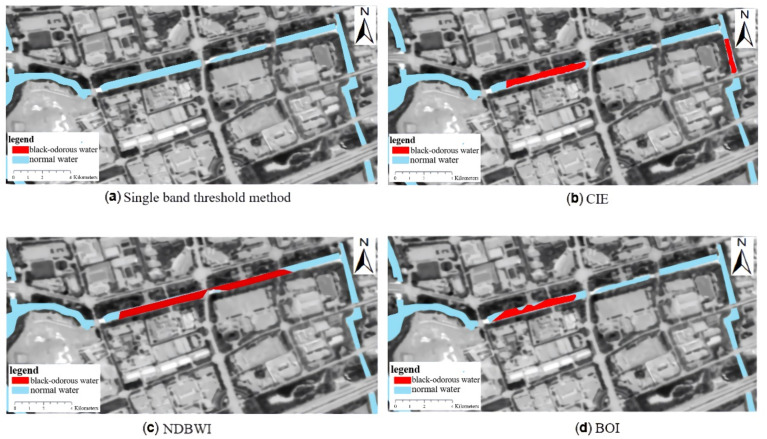
Recognition results of black-odorous water by different algorithms.

Based on the threshold values of the single-band threshold method, NDBWI, BOI, and CIE set by the modeling samples, the recognition accuracies were found to be 75%, 87.5%, 87.5%, and 50%, respectively. The river shown in Figure 10 is Dongchen River, where (a) represents the distribution of the black-odorous water extracted using the single-band threshold method from the remote sensing image, (b) represents the distribution of the black-odorous water extracted using the CIE chromaticity method from the remote sensing image, and (c) represents the distribution of the black-odorous water extracted by using NDBWI method from the remote sensing image. (d) Represents the distribution of the black-odorous water extracted using the BOI from the remote sensing image.

The single-band threshold method and CIE produces a low recognition rate for black-odorous water because of the following reasons: (1) In the green and red bands, the remote sensing reflectance values of the two water bodies are similar, and a single band cannot be used to distinguish the black-odorous water body from the general water body. (2) CIE is the judgment of the color of ground objects. The misjudgment is due to the different pollutants discharged into the river in the city, the different types of black-odorous water bodies, and the various colors of water bodies. The color of some of the water bodies is similar to that of ordinary water bodies, which not only increases the difficulty in distinguishing them, but also makes it easy to misjudge black-odorous water bodies as ordinary water bodies.

#### 3.2.3. Model Application

**Figure 11:**
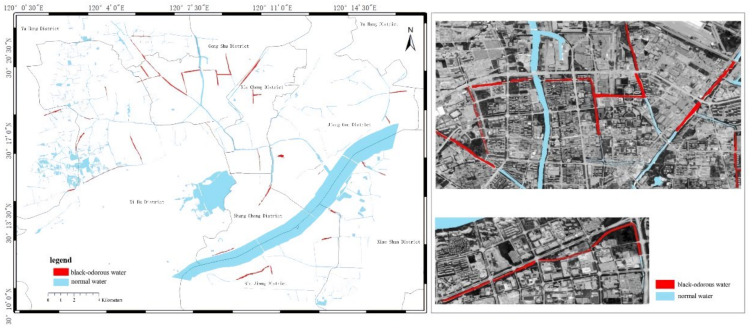
Distribution map of black-odorous water in 2015.

**Figure 12:**
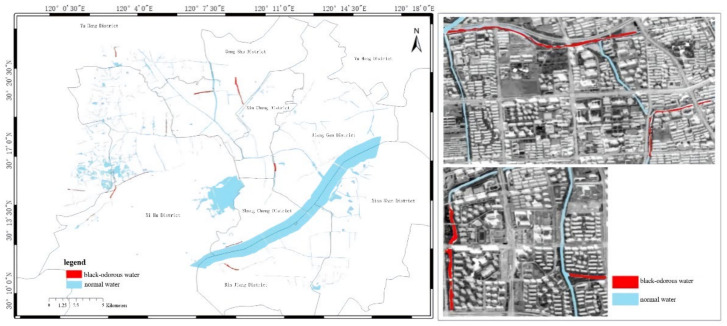
Distribution map of black-odorous water in 2016.

**Figure 13:**
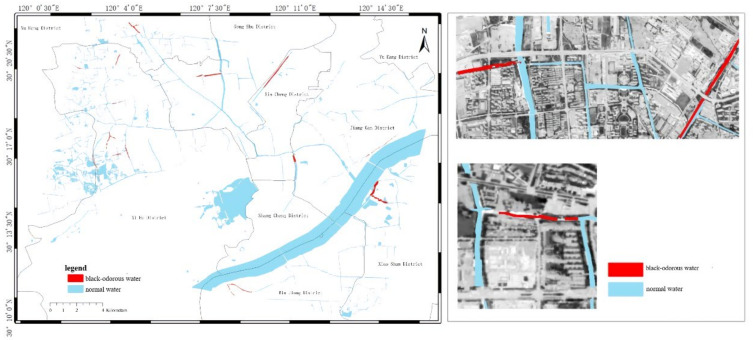
Distribution map of black-odorous water in 2018.

In modeling, the NDBWI has a higher recognition rate than the BOI method. Therefore, the NDBWI method was used to identify the black-odorous water bodies in the GF-2 images taken in 2015, 2016, and 2018; the recognition results are shown in the Figure 11, Figure 12 and Figure 13.

Temporally, the quantity of black-odorous water in 2016 decreased significantly compared with that in 2015. The quantity of black-odorous water in the Yuhang, Xiacheng, Jianggan, and Xihu Districts decreased; however, some of the water bodies in the Gongshu and Jianggan Districts still showed black-odorous phenomenon. In 2018, the number of black-odorous water bodies continued to decrease. The main problem of black-odorous water bodies lies in “darkening”, for example, some rivers in the downtown area have darkened.

Spatially, Gongshu and Xihu Districts in Hangzhou have the largest amount of black-odorous water, whereas Xiaoshan District has the least amount of black-odorous water. Black-odorous water bodies are mostly distributed in relatively narrow river channels, such as the Zhonghe river in the West Lake area and rivers near Wuchang Avenue. According to a field investigation, some black-odorous water bodies are located in residential areas on both sides of the river. For example, due to improper sewage treatment in nearby residential areas, the Shanqingzhuangheng River has oil floating on its surface. In the Houhenggang River, due to improper sewage treatment in the community, a part of the upstream water is black and odorous, and there is floating garbage. Nevertheless, the downstream part is far away from residential areas, and the water quality is good. Therefore, the same river has different black-odorous phenomena in different reaches. In addition, factories and construction sites are prone to black-odorous water bodies. For example, the Taojiawei river is under construction on both sides, and its water quality is poor.

## 4. Conclusions

(1) The remote sensing reflectance of black-odorous water was found to be lower than that of ordinary water (<0.04 sr^−1^), and the variation range of its remote sensing reflectance was narrower than that of ordinary water. In other words, the slope was low. In the visible and near-infrared band ranges, the peaks of the reflectance curve of black-odorous water showed a “red shift” phenomenon.”
(2) The single-band threshold method, CIE, NDBWI, and BOI were compared and analyzed. The NDBWI and BOI had the highest identification accuracy.
(3) The number of black-odorous water bodies in Hangzhou showed a decreasing trend, and there were no black-odorous water bodies in the upper city in 2018. The distribution of black-odorous water was concentrated in relatively narrow rivers with dense population and residential areas, factories, and construction sites on both sides. Moreover, different sections of the same river showed different water quality.
(4) The results of this study show that the area of the black-odorous water body in Hangzhou is decreasing year by year, which is consistent with the bulletin on the environmental condition of Hangzhou published by the Hangzhou Environmental Protection Bureau, proving that the water regulation action carried out in Hangzhou has achieved stable results. However, at the same time, the results also show that there is a “black” rebound in a few rivers, and the above results are consistent with the conclusions of Yu [32] et al.
(5) At the field level, there are few measured data and sample points, and some of the data is invalid due to weather and operation reasons, so the accuracy of the established model and the selected threshold is not high, and there is a certain error. In addition, the study area of this paper is small and does not cover the entirety of the water bodies in Hangzhou, which cannot represent the situation of black-odorous water in Hangzhou. In the aspect of internal operation, there is a certain time interval between the imaging time and the measurement time, so there may be a change in the reflectivity of the water body in a short time, resulting in data errors.
(6) Among all the models verified above, the NDBWI model and the BOI model are not only showing high accuracy, but are also convenient to use and fast in calculation. It is of great practical value for the rapid tracking and monitoring of urban black-odorous water for future work.

In this paper, when using this algorithm, due to the low remote sensing reflectance of some clean water, it is easy to distinguish this part of the water body as black-odorous water body. So this algorithm is not suitable for the discrimination of large clean water bodies and black-odorous water bodies.

For future research work, we will further increase the number of remote sensing images, analyze the temporal and spatial variation of black-odorous water in more detail, and finally develop batch processing of remote sensing images.

## Figures and Tables

**Table 1:** GF-2 satellite payload technical indicators.

Parameter	Indicators	
Spectral range	Panchromatic	450–900 nm
	Multispectral	Band 1: 450–520 nm
		Band 2: 520–590 nm
		Band 3: 630–690 nm
		Band 4: 770–890 nm
Spatial resolution	Panchromatic	1 m
	Multispectral	4 m
Bandwidth	45 km	

**Table 2:** Image information of the study area.

Imaging Time	Location of Image Center	Image Range
2 August 2015	lon: 120.136° E lat: 30.2498° N	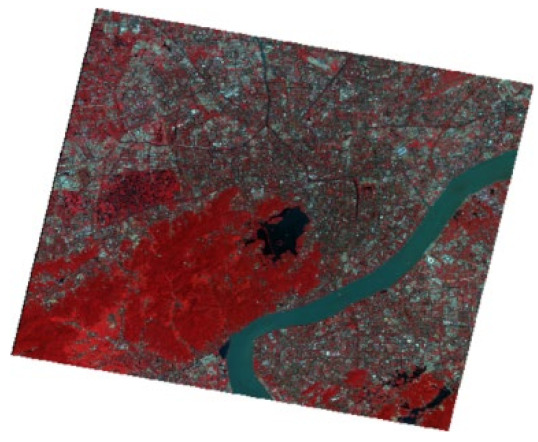
16 March 2016	lon: 120.292° E lat: 30.2505° N	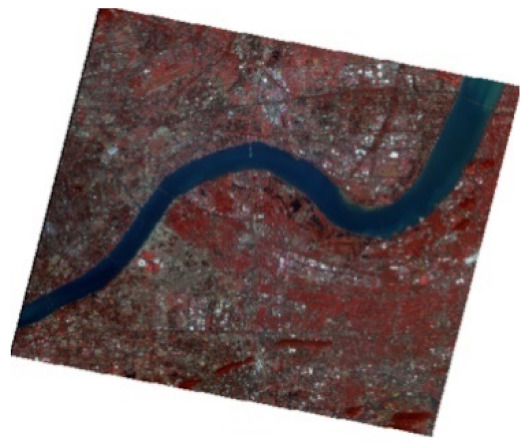
	lon: 120.067° E lat: 30.2937° N	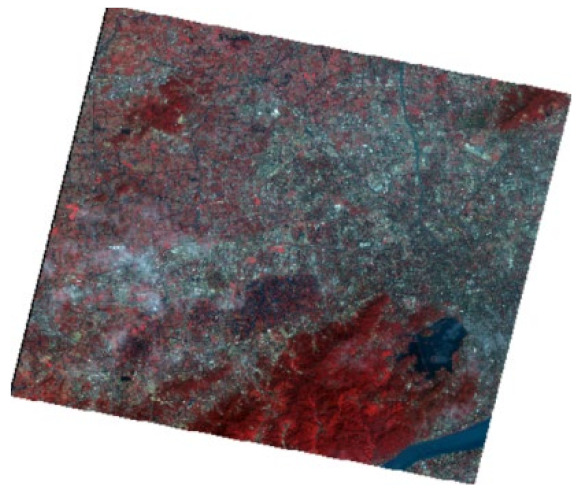
14 May 2018	lon: 120.232° E lat: 30.2453° N	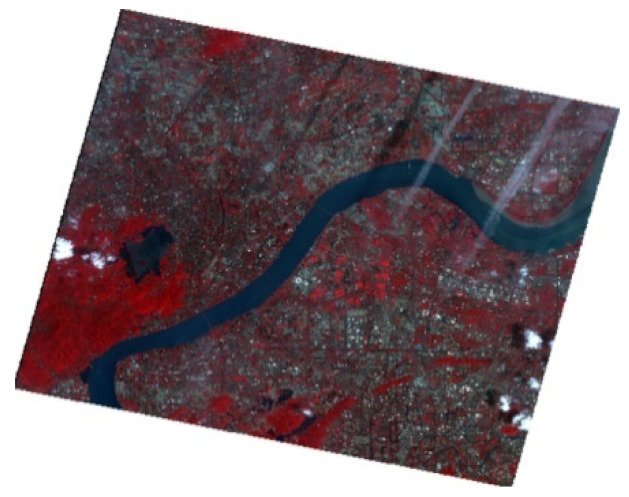
	lon: 119.974° E lat: 30.298° N	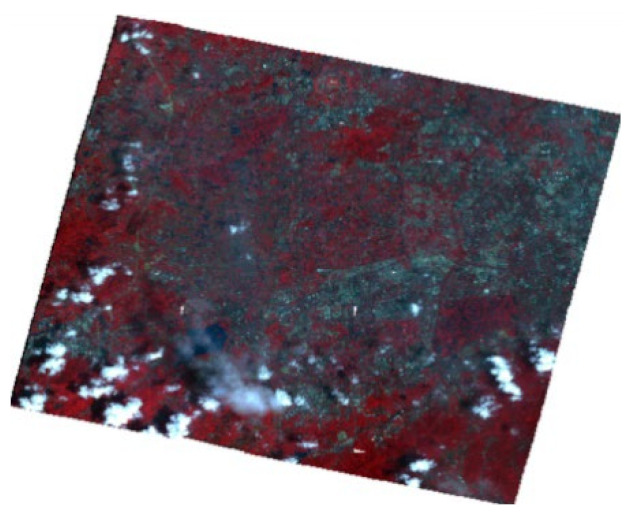

**Table 3:** DO content of black-odorous water body and general water.

Water Type	DO Content (mg/L)
Black-odorous water	3.4028
General water	7.0950

**Table 4:** Accuracy evaluation.

Sample Name	Single-Band Threshold Method		NDBWI		BOI		CIE		Actual Water Quality
	Calculation Results (sr^−1^)	Identification Results	Calculation Results	Identification Results	Calculation Results	Identification Results	Calculation Results (nm)	Identification Results	
P_1_	0.0539	normal	0.1333	normal	0.087	normal	504	normal	normal
P_2_	0.0187	odorous	0.1353	normal	0.096	normal	550	normal	odorous
P_3_	0.0168	odorous	0.2036	odorous	0.144	odorous	542	normal	odorous
P_4_	0.0362	odorous	0.1681	odorous	0.109	odorous	502	normal	odorous
P_5_	0.0409	normal	0.1615	odorous	0.125	odorous	537	odorous	odorous
P_6_	0.0346	odorous	0.1240	normal	0.096	normal	499	normal	normal
P_7_	0.0667	normal	0.1383	normal	0.090	normal	500	normal	normal
P_8_	0.0152	odorous	0.2303	odorous	0.183	odorous	558	normal	odorous

## Data Availability

Not applicable.
